# Chemical characterization of pterosaur melanin challenges color inferences in extinct animals

**DOI:** 10.1038/s41598-019-52318-y

**Published:** 2019-11-04

**Authors:** Felipe L. Pinheiro, Gustavo Prado, Shosuke Ito, John D. Simon, Kazumasa Wakamatsu, Luiz E. Anelli, José A. F. Andrade, Keely Glass

**Affiliations:** 1 Laboratório de Paleobiologia, Universidade Federal do Pampa, São Gabriel, 97300-162 Brazil; 2 Instituto de Geociências, Universidade de São Paulo, São Paulo, Brazil; 3 Department of Chemistry, Fujita Health University School of Medical Sciences, Toyoake, Aichi 470-1192 Japan; 4 Lehigh University, Bethlehem, PA 18015 USA; 5 Centro de Pesquisas Paleontológicas da Chapada do Araripe, Departamento Nacional de Produção Mineral, 63100-440 Crato, Brazil; 6 Department of Chemistry, Duke University, Durham, NC 27708 USA

**Keywords:** Palaeontology, Palaeontology

## Abstract

Melanosomes (melanin-bearing organelles) are common in the fossil record occurring as dense packs of globular microbodies. The organic component comprising the melanosome, melanin, is often preserved in fossils, allowing identification of the chemical nature of the constituent pigment. In present-day vertebrates, melanosome morphology correlates with their pigment content in selected melanin-containing structures, and this interdependency is employed in the color reconstruction of extinct animals. The lack of analyses integrating the morphology of fossil melanosomes with the chemical identification of pigments, however, makes these inferences tentative. Here, we chemically characterize the melanin content of the soft tissue headcrest of the pterosaur *Tupandactylus imperator* by alkaline hydrogen peroxide oxidation followed by high-performance liquid chromatography. Our results demonstrate the unequivocal presence of eumelanin in *T. imperator* headcrest. Scanning electron microscopy followed by statistical analyses, however, reveal that preserved melanosomes containing eumelanin are undistinguishable to pheomelanin-bearing organelles of extant vertebrates. Based on these new findings, straightforward color inferences based on melanosome morphology may not be valid for all fossil vertebrates, and color reconstructions based on ultrastructure alone should be regarded with caution.

## Introduction

Fossilization is a rapid process that degrades and converts the biomolecules that define the characteristics of living organisms into long, nearly indistinguishable chains of stable hydrocarbons^1,2^. Melanins, however, demonstrate surprising resilience in the geological record due to their polymeric, highly cross-linked structures^3–6^. Widely distributed within vertebrates as one of the main colour-producing biochromes, melanins constitute a class of heterogenous molecules derived from L-tyrosine^6,7^. In animals, melanins are found either as eumelanins, associated to dark brown/black hues, or phaeomelanins, which correspond to pale yellow to rufous brown tones^8^. In vertebrates, melanins are synthesized and stored in specialized organelles called melanosomes, which are usually found in integuments (and its appendages) as well as in internal organs^9^. Melanosomes are fairly common in exceptionally preserved fossils as 200–2000 nm long microbodies, and are generally associated with keratinized soft tissues, such as feathers and hairs^10–12^. Melanosome morphology is often used as a proxy for animal color, so the presence of these organelles in the fossil record has broad biological implications^11,12^.

The morphological similarities between melanosomes and exogenous bacteria means that the observation of microbodies found in fossilized soft tissues does not guarantee the preservation of pigments^13–18^. On account of this, direct chemical protocols are necessary for the unequivocal identification of these microbodies as preserved melanosomes.

**Figure 1:**
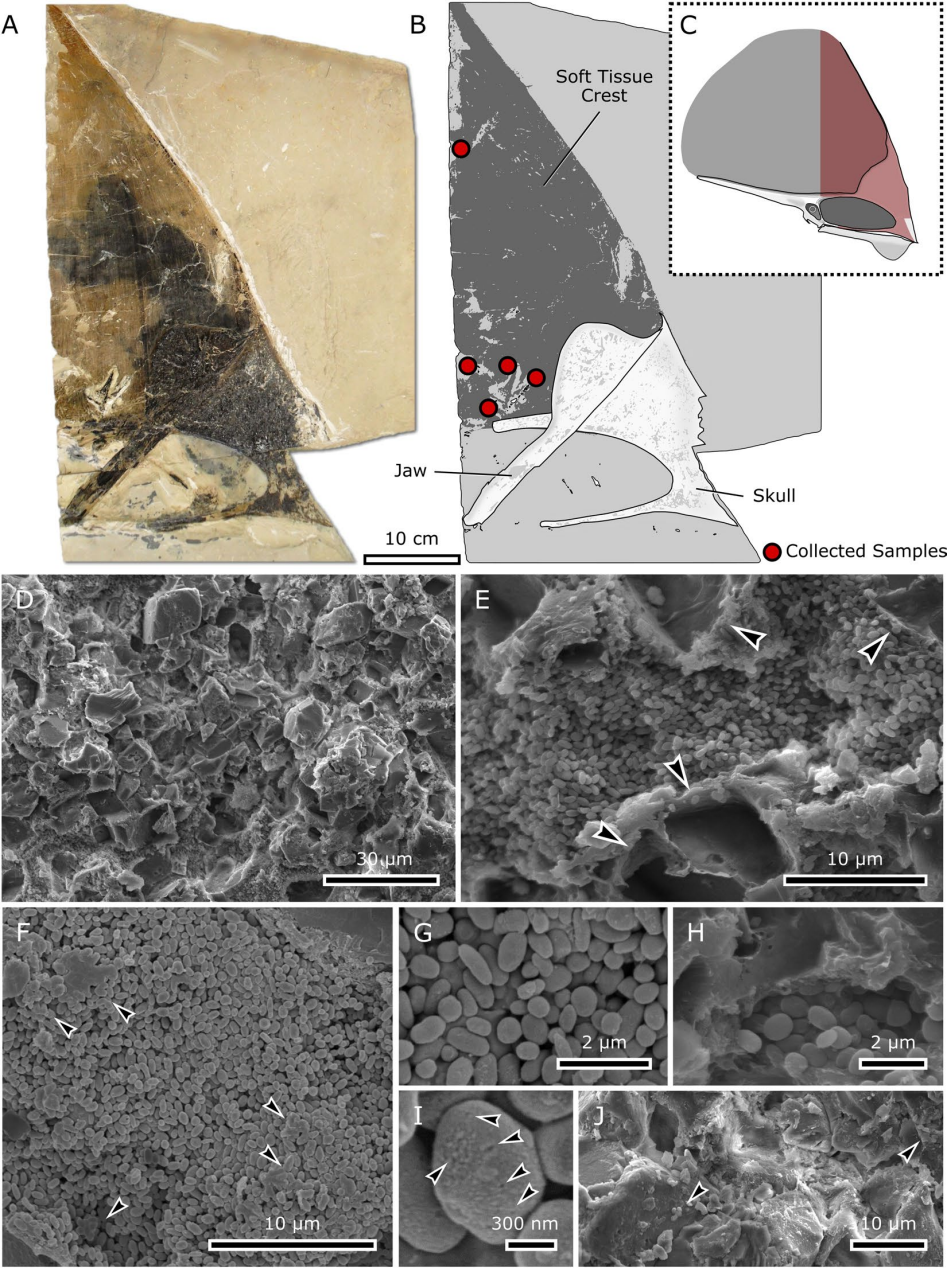
*Tupandactylus imperator* (specimen CPCA 3590) from the Crato Formation and headcrest microbodies. (**A**) Partial skull with its lower jaw overlying the headcrest. (**B**) Interpretative drawings of the figure (**A**,**C**) according to skull position. (**D**) Headcrest tissue exhibiting the blocky calcite crystals, and (**E**) melanosomes amidst external molds of neomorphic crystals (arrowheads). (**F**,**G**) Densely packed microbodies with subspherical morphology. (**H**) Keratin-like structure overlying or surrounding particles. (**I**) Scattered pits (arrowheads) on the microbody surface. (**E**) Calcite crystals blocks located amongst microbodies, with several pigmentary particles scattered on their surface (arrowheads).

Pterosaurs were a diverse group of Mesozoic flying archosaurs, which usually borne conspicuous cranial ornamentation in the shape of the bone or soft tissue headcrests. Pterosaur headcrests display strong positive allometric growth^19^ and are sexually dimorphic traits in some species^20^, which support their function as display structures^21,22^. Here, portions of the Brazilian pterosaur *Tupandactylus imperator* headcrest (Fig. 1A–C) were degraded and analyzed using alkaline hydrogen peroxide oxidation and high-performance liquid chromatography. Samples of the crest tissue were also analyzed using Raman Spectroscopy and Synchrotron Radiation X-Ray Fluorescence (Supplementary Information). Our results are the first to demonstrate the presence of preserved melanin in an archosaur and challenge color inferences in extinct animals using melanosome morphology alone.

## Results

### Scanning electron microscopy (SEM)

While limited to the headcrest, microbodies exhibit a wide distribution throughout the tissue, albeit forming local clusters (Fig. 1D). They occur amidst neomorphic crystals rather similar to “blocky calcite crystals”^23^ (Fig. 1E). There is also evidence of lamellar minerals typical of clays, such as phyllosilicates.

The microbodies are densely packed (Fig. 1F,G), with an average size of 441 ± 96.5 nm in diameter and 653 ± 148 nm in length (n = 331), indicating that their morphology is predominantly prolate (Supplementary Information). The microbodies retained their integrity without being warped or broken throughout the imaged region, with the exception of a small portion of microbodies. The scattered pits on their rough surfaces, occurring in several parts and indiscriminately to morphology, are attributed to the 10 nm layer of Au/Pd coating applied to the surface to increase spatial resolution (Fig. 1I). In highly dense regions, microbodies are also attached to an amorphous structure (Fig. 1F), but these are much rarer than the free microbodies. Particles can also be found onto blocky crystals, where they occur in small bundles (Fig. 1J).

### Raman spectroscopy (RS)

**Figure 2:**
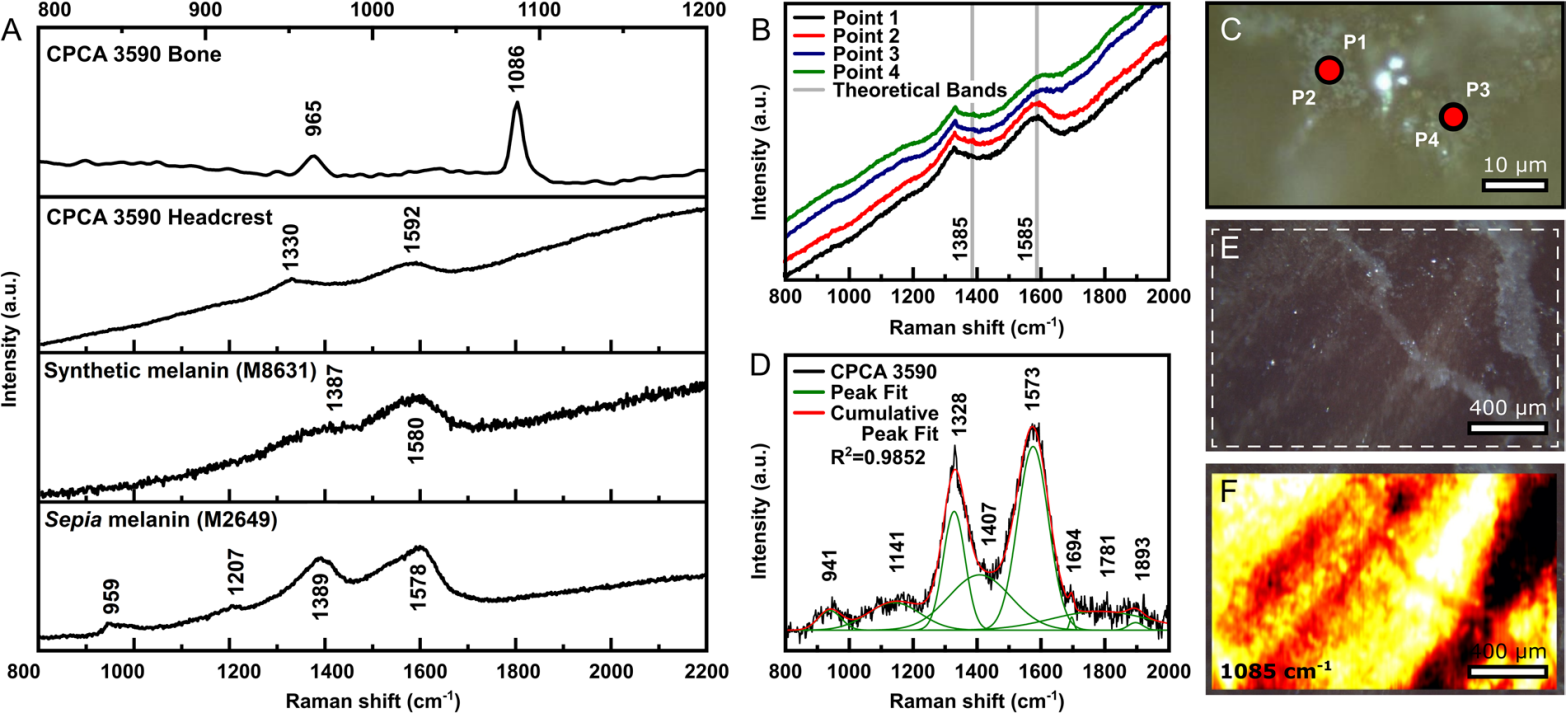
Raman spectra from *T. imperator* (CPCA 3590) headcrest and melanins. (**A**) The top spectrum is from the bony area of the headcrest showing peaks of CO_3_^2−^ (at 1086 cm^−1^) and PO_4_^3−^ (at 965 cm^−1^), which is consistent with a bioapatite variety. The second spectrum is that of the headcrest, and is similar to the two shown below, those of synthetic and *Sepia* melanins, respectively. (**B**) Raman spectra from dark bands of the headcrest exhibiting a signal variation between the two regions of measurement (red circles indicate the two points where four measurements were made) (**C**). Grey dashed lines in (B) represent the theoretical bands of eumelanin. (**D**) Fitted spectra using Gaussian function (*R*^2^ = 0.9852) from the Point 1 seen in (**B**), showing that multiple bands are also observed as predicted in other studies. (**E**) Microscopic image from the headcrest surface, showing the region where the fluorescence mapping was performed (white dashed lines). (**F**) Map of the 1085 cm^−1^ region that is diagnostic of calcite, from the area seen in (**E**), indicating a faint signal of the soft tissues, suggesting that calcite from matrix predominates.

The Raman spectra of *T. imperator* headcrest contain diagnostic peaks both of calcium phosphate and eumelanin (Fig. 2A; Fig. S4)^24–31^. Although the peaks for apatite and eumelanin are present throughout the tissue, the associated bands are more intense in the dark stripped regions of the headcrest (Fig. 2B,C; Fig. S4, A, B). Based on the presence of 318 cm^−1^ and 1077 cm^−1^ peaks (Figs 2E,F and S4C), comparative analysis with standard minerals indicates that CPCA 3590 bone and soft-tissues consists of hydroxyapatite (Ca_5_(PO_4_)_3_(OH)). More important, the identified bands at *ca*. 1330 to 1592 cm^−1^ are in overall agreement with the diagnostic spectra of eumelanin^24–31^.

The fitting of CPCA 3590 spectra (Fig. 2D) yielded several bands but the most diagnostic ones occur centered at about 1328 cm^−1^ and 1575 cm^−1^ (Table S2). Despite the former is slightly shifted to the left, both bands are similar to those of synthetic and *Sepia officinalis* melanins (Sigma-Aldrich M2649) from our experiments (Fig. 2A) and reported in the literature. For synthetic melanin (Sigma-Aldrich M8631), the two most intense peaks occur at 1387 cm^−1^ and 1580 cm^−1^, whereas for the *Sepia*-derived melanin, they are centered at about 1389 cm^−1^ and 1578 cm^−1^. Other compounds present in the sample, such as carbonates and phosphates, may be influencing the spectra and affecting the melanin peak intensities (Fig. 2E,F). Furthermore, the broad bandwidth is reflective of the heterogeneity/disorder of eumelanin structure^24^, which may have incorporated metals, especially Ca and Mn, among its oligomer sheets^32^. Moreover, the slight shift in the Raman peaks may also indicate that *T. imperator* eumelanin went through a substantial change, possibly related to loss of functional groups, or may also be derived from the C–N stretching from the indole ring^33^. Regardless of these features, both bands can be confidently assigned to the stretching and plane vibrations of C–C, C–OH, C–N, C–O from pyrrole and indole rings^25,27–29,31,33^ (Supplementary Information). The less intense peaks in CPCA 3590 may result from the trace amounts of melanin. In contrast to carbon-rich compounds that exhibit overtone scattering bands (second-order peaks) above 2400 cm^−1^ ^34^, these peaks are absent, supporting the argument that they are derived from eumelanin^28,29^. The previous interpretation of phosphatization of the headcrest and microbodies^35^ is also supported by RS.

### Identification of eumelanin by chemical degradation and high-performance liquid chromatography (HPLC)

The first modern attempt to chemically characterize melanins in the fossil record used synchrotron X-ray to identify trace metals alleged to be unique markers of the presence of eumelanin^36^. Since then, several attempts have been made using this methodology to evaluate different types of fossils^37^. However, this method did not survive scrutiny, as several taphonomic processes are able to concentrate metals and originate similar patterns under synchrotron light sources^13,37^. More recently, chemical fingerprints identified by time-of-flight secondary ion mass spectrometry (ToF-SIMS) was successfully employed to characterize both pheo- and eumelanins in fossils^4,10,15,16,38–40^. Direct and conclusive chemical evidence, however, requires carrying out chemical degradation of the organic matter by alkaline hydrogen peroxide oxidation, which, if melanin is present, generates specific and unique chemical markers^3,41,42^.

Specifically, pyrrole-2,3,5-tricarboxylic acid (PTCA) and pyrrole-2,3-dicarboxylic acid (PDCA) are the chemical degradation markers of 5,6-dihydroxyindole-2-carboxylic acid (DHICA) and 5,6-dihydroxyindole (DHI), respectively, and, hence, of eumelanin^41^. Similarly, pyrrole-2,3,4,5-tetracarboxylic acid (PTeCA) is considered an index of highly cross-linked, “aged” melanin^5^. As a consequence, it is suggested that PTeCA must be the most common moiety in ancient materials that underwent mild thermal maturation^42^.

**Figure 3:**
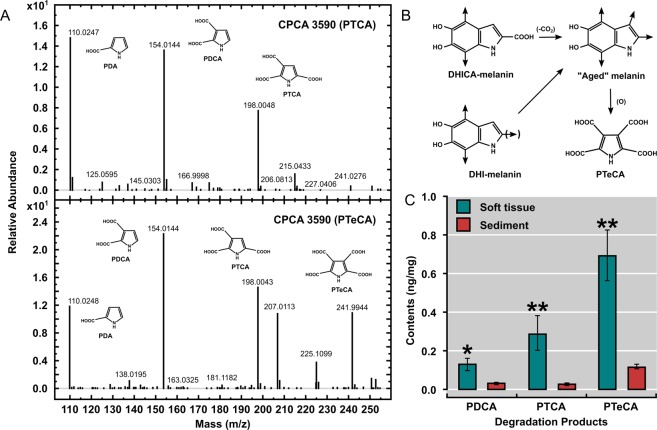
Mass spectrum of CPCA 3590 headcrest melanin. (**A**) Mass spectrum from the degradation products of alkaline peroxide oxidation. (**B**) Hypothetic structural modifications of eumelanin from *T. imperator* (based on Ito *et al*.^5^), according to the most abundant yielding recovered from the oxidation process (i.e. PTeCA). (**C**) Oxidation was performed on four separate occasions and the results were shown as mean ± SEM. Values for the sediment are close to the detection limits. Values are in ng/mg. Differences as evaluated by students’ t-Test (two-tailed) are *P* < 0.01 except for PDCA (*P* < 0.05).

In this study, samples of *Tupandactylus imperator* headcrest (CPCA 3590) were oxidized by alkaline hydrogen peroxide after demineralization, in accordance to standard protocols^3,41,42^ (Materials and Methods; Supplementary Information). HPLC analysis of the oxidation products yielded melanin markers PTCA, PDCA, and PTeCA (Figs 3, S4, Table S3). Although their levels were trace, they were significantly higher than those in the adjacent sediment, and their identification was also confirmed by liquid chromatography-mass spectrometry (LC-MS, See Fig. S5). It is noteworthy that the level of PTeCA is much higher than that of PTCA, with a PTeCA/PTCA ratio being 2.41 ± 0.16, which is characteristic of highly cross-linked eumelanin^3,5^. Ergo, the results of HPLC (Fig. 3A) indicate that most of the *T. imperator* eumelanin is derived from the crosslinking of DHICA and DHI fractions (Fig. 3B). Although the contribution of PDCA from DHI units cannot be excluded, because PTCA occur twice as much (Fig. 3C), it is suggested that this moiety is more involved in structural alterations^5,42^.

Pheomelanin oxidation produces thiazole-2,4,5-tricarboxylic acid (TTCA) and thiazole-4,5-dicarboxylic acid (TDCA); both markers are derive from the pigment’s benzothiazole moiety^5,41^. As neither marker were detected in this samples, the predominant pigment in the CPCA 3590 headcrest is eumelanin.

## Discussion

Previous microstructural analyses of CPCA 3590 identified the subspherical microbodies as autolithified bacteria^35^, an assumption mainly based on (i) their comparable size with modern microorganisms; (ii) the lack of the typical organization patterns often seen in melanosomes; (iii) the presence of supposed extracellular polymeric substances and (iv) the putative ongoing cellular division^35^. This interpretation was debated in subsequent publications – questioned by some^43^ and favored by others^13–16^. As pointed out by the authors that favored the bacterial alternative, the physical aspects of these microbodies, such as morphology, distribution, and size, were insufficient to completely eliminate the hypothesis of both endogenous or exogenous bacteria^17,18,44–46^. However, it is widely accepted that chemical analysis can significantly aid in their identification^10,13–16,45,47,48^.

Although microbes are rarely preserved, the fossilization of animal soft-tissues usually involves the presence of microorganisms that alter geochemical processes at the microscopic level, inducing the precipitation of several minerals^44,49,50^. Experiments simulating diagenesis in microbes indicate that although their molecular signatures can be slightly altered^51^, their morphology often exhibits significant changes, mostly in the form of body deflation or partial degradation^52^. These features are absent in our sample, in which microbodies are predominantly solid particles. Furthermore, *T. imperator* microbodies also show several characteristics that are consistent with being melanosomes, such as absence of morphotype diversity, no evidence of binary fission and lack of bacterial by-products (such as honeycomb-like structures)^53^, distinct chemistry differences in chemical composition between former soft tissue and matrix^54^, and limited microbody distribution (Supplementary Information).

Energy dispersive spectroscopy (EDS) data show that *T. imperator* microbodies are Ca- and P-rich, suggesting that they are composed of calcium phosphate^35^. In addition, SR-μXRF indicates the presence of Ca, Cu, Fe, Mn and Zn (Fig. S3). Since phosphatization is the common type of bacterial preservation^50^, these microbodies could indeed represent phosphatized microorganisms. However, the chemical signatures revealed in our study show that the CPCA 3590 headcrest contains eumelanin. Therefore, the combination of morphological and chemical analyses confirms an unequivocal identification of the microbodies as melanosomes.

Since the seminal work of Vinther *et al*.^55^, inferences about the color patterns of fossil animals rely mainly on melanosome morphology^13^, in spite of other studies that indicate the lack of correlation between melanosome shape and their melanin content^56,57^. While the connection between shape and color is unresolved^13–15,58^, it is commonly invoked that high-aspect-ratio (“sausage-like”) melanosomes contain eumelanins (black to dark brown in color), whereas globular, low-aspect-ratio melanosomes normally reflect the presence of pheomelanin (rufous red to pale yellow). Moreover, statistical analyses testing the correlation between melanosome morphology and color of extant birds demonstrated a high (up to 82% accuracy) predictive potential for animals in which hues are mainly determined by melanins^13,59,60^.

A recent contribution^59^, however, demonstrated that a similar predictive model cannot be extrapolated to lepidosaurs, turtles, and crocodiles, whereas it is reasonably accurate for bird feathers and mammalian hair. As such, these latter animals would present a high diversity of melanosome morphologies and usually a clear correlation between different morphotypes, the type of melanin they contain and, as a consequence, expressed color^59^. The transition between the primitive low melanosome diversity displayed by lizards, turtles and crocodiles and the pattern displayed by present-day mammals and birds would have been driven by a distinct physiological shift^59^. Alternatively, this change in pattern would be a consequence of the loss of the chromatophore complex, responsible for the color diversity of amniotes showing the primitive condition^13^. The chromatophore system might be superfluous for animals in which color patterns are expressed in well-developed integumentary structures, such as feathers and fur^13^.

It would, however, be expected that pterosaurs did not depend on chromatophores to express color patterns, as these archosaurs were also covered by a dense layer of supposedly keratinous filamentous structures that were potentially homologous to feathers^61^. The analyses of Li *et al*.^59^ included two pterosaur specimens, in which the microstructure of the hair-like coverage showed a low diversity of low-aspect-ratio melanosomes, more consistent with what is observed in lepidosaur, turtle and crocodile skin than to feathers or mammal fur. A similar pattern is also displayed in *Tupandactylus imperator* headcrest (based on CPCA 3590). Morphologically, the vast majority of the microbodies revealed by SEM images would be identified as pheomelanin-like melanosomes. In spite of that, the chemical degradation performed yielded the specific markers of eumelanin (i.e. PTCA, PDCA, and PTeCA) in concentrations compatible with highly cross-linked eumelanin^3,5,42^, and the absence of the specific markers for pheomelanin. Thus, these results imply that a clear distinction between high-aspect-ratio eumelanosomes and spherical phaeomelanosomes is not valid for pterosaurs, and by extrapolation, for amniotes that share the primitive condition of low melanosome diversity. Indeed, CPCA 3590 organelles are remarkably similar to internal eumelanosomes^58^ from basal-most vertebrates, such as the amphibians, cyclostomes^38,39^ and cuttlefish^3^. Consequently, any color inference in animals presenting the plesiomorphic condition based on melanosome morphology would be equivocal, as ellipsoidal, low-aspect-ratio bodies can contain both pheo- and eumelanins. We should also stress that the circumstances surrounding the physiological shift proposed by Li *et al*.^59^ are still obscure, and it would be precipitate to imply that animals such as non-avian dinosaurs shared with birds a straightforward correlation between melanosome morphology and their pigment content.

Most chemical surveys of fossil pigments have thus far identified eumelanosomes and eumelanin fingerprints. The reason for the low occurrence of pheomelanin or pheomelanosomes is still unknown; it remains possible that pheomelanin preservations may not be as robust as that of eumelanin^62^. Although other classes of biochromes (e.g. carotenoids and porphyrins) are relatively common in sedimentary deposits, these compounds are extremely frail and prone to chemical alterations^1,63,64^. For instance, following deposition, porphyrins, and carotenoids readily experience several chemical reactions such as oxidation and polymerization, transforming them into long chains of hydrocarbons^1,65,66^. Because melanin is a highly conserved polymer^5,10^, and eumelanins are the most common class of melanins in nature, it is expected that this pigment is present in the majority of exceptionally preserved fossils^12,13^.

Studies that identified pheomelanin have rarely found corresponding microbodies preserved in three dimensions. As a consequence, phaeomelanic colorations were often based on the recognition of melanosome external molds^67^ or chemical fingerprints^40^. Despite the latter approach being a more reliable way to identify pheomelanin, the former possesses serious issues to color inferences. Our results support this claim, as spherical and subspherical microbodies can potentially bear one or both types of melanin pigments or be composed mainly by one type of moiety. This may be true to some dinosaurs, such as *Sinosauropteryx*^67^, *Anchiornis*^68^, *Yi qi*^69^, and *Psittacosaurus*^70^, whose color inferences were based solely on the morphology of molds, with no further chemical and/or statistical support.

The extremely selective nature of fossilization has the effect of building a virtually insurmountable barrier between post-diagenetic remains of organisms and living beings. The recent recognition of the persistence of melanins and melanin-containing organelles in the fossil record^3,42^ allowed reconstructions of color patterns of extinct animals. However, many paleocolor studies relied basically on the microbody morphology, raising questions about the validity of their outcomes. Correspondingly, our results strongly support these disputes. Since melanins are directly involved in complex social and ecological behaviors, such as camouflage, intraspecific recognition, and sexual display, their correct characterization can sum to the understanding of the biology of extinct animals^13^, and color reconstruction cannot rely solely on microstructural analysis^13,71^.

## Materials and Methods

Specimen CPCA 3590 is preserved in a grayish-color laminated limestone typical of Crato Formation beds^35^ (Supplementary Information). This fossil is comprised of a fairly complete skull with headcrest’s soft tissues, which allows it to be assigned to the tapejarid species *Tupandactylus imperator* (for taxonomic details, see Pinheiro *et al*.^70^). This specimen is permanently housed in the Paleontological collection of the *Centro de Pesquisas Paleontólógicas da Chapada do Araripe* (CPCA, Crato, Ceará, Brazil). The headcrest’s soft tissue was examined using a scanning electron microscope (SEM). Elemental mapping was carried out using synchrotron radiation-micro X-ray fluorescence (SR-µXRF). The molecular content was examined using Raman spectroscopy (RS) and high-performance liquid chromatography (HPLC). The latter technique was performed to quantitate melanin degradation products, PTCA, PDCA, and PTeCA after treatment by alkaline hydrogen peroxide oxidation of demineralized samples of CPCA 3590^5,41^. To confirm the identification of PTCA and PTeCA, LC-MS of extracts of oxidation products was performed according to previously described methods (see Glass *et al*. 3). Following the image acquisition using SEM, melanosomes and minerals were measured using ImageJ^72^, and statistical analysis was performed using Past 3.06^73^. SR-µXRF mapping was processed using PyMCA 5.1.1 software and Raman spectra were processed using Renishaw Wire 4.1 and Wire 4.4, and Origin 9.6.0.172. Analyses were performed at the Duke University Chemistry Department Mass Spectrometry Facility, Brazilian Synchrotron Light Laboratory (LNLS) and Institute of Chemistry of the University of São Paulo (IQ-USP). See SOM2 for details on material and methods.

## Supplementary information

Supplementary Information

## Data Availability

No datasets were generated or analyzed during the current study.
