# Supplementary material for: Chemical characterization of pterosaur melanin challenges color inferences in extinct animals
Source: Sci Rep. 2019 Nov 4;9:15947. doi: 10.1038/s41598-019-52318-y (PMC6828676; doi:10.1038/s41598-019-52318-y)
Supplement: Supplementary file 1 — Supplementary Information [file 41598_2019_52318_MOESM1_ESM.docx]

Supplementary Information for

**Chemical characterization of pterosaur melanin challenges color inferences in extinct animals**

Felipe L. Pinheiro; Gustavo Prado; Shosuke Ito; John D. Simon; Kazumasa Wakamatsu; Luiz E. Anelli; José A. F. Andrade; Keely Glass

Felipe L. Pinheiro

Email: felipepinheiro@unipampa.edu.br

**This PDF file includes:**

Supporting methods

Figures S1 to S5

Tables S1 to S3

**1. Supporting text**

- 1. **Geologic Setting**

Within the geological succession of the Araripe Basin, Northeastern Brazil (Fig. S1, A), the Crato Formation is one of the four Lower Cretaceous units of the Santana Group ^1^, the others being the Barbalha, Ipubi and Romualdo formations ^2^. The Crato Formation is limited to the N-SE portions of the basin, more conspicuously in the Cariri Valley, Ceará State. Lithologically (Fig. S1, B), this unit consists of laminated limestones and greenish shales sequences with virtually the same thickness, which are also interbedded by sandstones and, occasionally, by thin layers of evaporitic minerals, predominantly gypsum.

The Crato Formation limestone is composed of fine-grained calcite crystals (micrite and spate), with varying content of amorphous organic matter and siliciclastic minerals ^3–5^. According to some authors, the carbonate-siliciclastic sequences were deposited in a protected lacustrine environment, with a strong chemocline gradient, especially with respect to salinity and oxygen concentrations ^3,6^. Other authors however argue that only the lowermost beds of the Crato Formation (i.e. Nova Olinda member *sensu* (6), which is the thicker carbonate sequence, can be considered as lacustrine ^2^. All other sedimentary series may represent a palaeolake with direct contact with seawater, Sabkha or lagoon environment ^2^. The presence of marine bivalves at the top of Crato Formation succession seems to support this interpretation ^8^.

Independent of the environment, the genesis of the Crato Formation limestones is related to authigenic precipitation of low-Mg calcite. Crystals were precipitated following seasonal phyto- and picoplankton blooms ^5^, seasonal salinity fluctuations caused by evaporation ^7^, and/or induced by bacteria from microbial mats and stromatolites ^9,10^. This is supported by the presence of fossilized calcified bacteria reported in the region (see Catto et al., 2016 and Warren et al., 2017) as well as components that are directly associated with the microbial activity, such as honeycomb-like structures and pseudomorphs of pyrite ^11,12^. Therefore, these microorganisms are considered a determinant for exceptional preservation of Crato Formation fossils ^13^. Different diagenetic processes occurred in the different facies in which fossils are confined. Non-recalcitrant tissues were replaced by iron sulphides at beige facies, whilst kerogen and calcium phosphates predominate in the greyish facies ^11,14,15^. The abundance of fresh water parautochthonous fauna (as Ephemeroptera larvae and anurans) in association to halite pseudomorphs indicates fresh shallow waters at the top, with a putatively episodic hypersaline bottom ^5,7,10^. Similarly, the absence of benthic fauna and bioturbated sediments indicate that deep waters were anoxic ^7^.

- 1. **Statistical analysis**

A statistical survey of size distribution indicates that 11.5% (n=38) of melanosomes lengths are around 576 nm, whereas 15.7% (n=52) of the diameter falls around 442 nm. The aspect ratio distribution indicates that 16% (n=53) are concentrated around 1.1, suggesting that most melanosomes are more oblate than cylindrical. These results considerably contrast with descriptive values, such as mean, standard deviation and median (Table S1). As expected, the size distribution does not correspond to mean values, since the latter only indicate the mean value of the whole population.

Performed scatter plot analysis shows a weak correlation between length and diameter (Fig. S2, A; *r*=0.4084; *R^2^*=0.1668), and One-Way ANOVA results showed to be statistically significant (*p*<0.001), indicating that both axes are indeed independent variables. This interpretation is also supported by the ratio frequency (Fig. S2, B), which exhibits a mean of 1.5 ± 0.4, while 74.3% of the total ratio occurs between 1.1 to 1.7 frequencies. This result is expected, since these units are usually considered independent variables, and the low correlation and frequency values indicate that CPCA 3590 melanosomes are predominantly oblate/oval. We performed a principal component analysis (PCA) using both CPCA 3590 and the database provided by Li et al. ^16^ (Fig. S2, C). As a result, the CPCA 3590 microbodies are placed close to melanosomes from brown and penguin colours (i.e. dark black). Size correction does not have a significant impact on this relationship but, at 20%, it brings much closer to penguin (blackish) coloration. On the other hand, melanosomes are close to brownish hue in the bulk data.

**1.3. Synchrotron Radiation X-Ray Fluorescence (SR-μXRF)**

Despite general scepticism of the usefulness SR-µXRF for melanin characterization (cf. 2), we performed an elemental mapping of small samples of CPCA 3590 (Fig. S3). This method aimed to identify elemental distribution throughout the specimen and relate the presence of particular ions (e.g. sulphur) to the process of fossilization and melanogenesis ^18^. Albeit different regions were examined, and other elements were found, these results served as a complement to the EDS spectra published in Pinheiro et al. (24: Fig. 3). As a result, the elemental mapping of CPCA 3590 exhibits the presence of As, Ca, Cu, Fe, Mn, Sr and Zn in headcrest tissue in varying intensities (Fig. S3). For instance, Ca and Mn display a more pronounced and spatially limited occurrence, whereas As, Cu, Fe, Sr and Zn are less intense and more disperse, reflecting their possible minor concentration in the sample.

Several works have demonstrated the usefulness of SR-μXRF on the elemental mapping of fossils ^20–23^, even when elements are present in trace amounts ^24–27^. In addition, this method has proved its usefulness for the identification of metals associated with melanin preservation ^20,28^. A recent study was able to positively identify the distribution of trace elements allegedly reminiscent of melanins in extant feathers ^28^. Despite being recognized in trace amounts, we were able to identify elemental distribution in CPCA 3590 samples. Following the analyses, we examined possible sources for the identified elements in *T. imperator* headcrest.

The intense presence of calcium is considered here as being mainly derived from matrix carbonate. Moreover, it is also possible that lower concentrations might be derived from the fossil itself, since this ion can be found in apatite ^29^ or incorporated into melanin ^30^. On the other hand, the source of manganese might be allochthonous, as this mineral is usually found in its oxidized pyrolusite (MnO_2_) form ^31^. Indeed, this mineral has already been reported in the Crato beds as a component of dendritic habits that are sometimes associated with fossils ^7^. Since copper, iron, and zinc are essential to some physiological processes, the presence of these elements is often considered as autochthonous (or parautochthonous in the case of Fe), is derived from the animal itself or its environment ^21^. Some authors also suggest that, because Cu, Zn, and S have high affinity to melanin, these elements may form organic chelates which in turn may serve as markers for this pigment ^32^. However, sulphur was not identified in our SR-μXRF analysis, and Cu and Zn can be also incorporated by microbial activity or during diagenesis.

We note that some of these interpretations are merely speculative since experiments to recognize element oxidation states, such as X-ray absorption near edge structure (XANES) are still needed. As such, these elements could be also derived from the environment, from the preserved organic matter, or eumelanin. We acknowledge that at least three possible causes might explain the presence of the trace metals, as follow: (i) endogenously derived, since they are involved in physiological processes; (ii) accumulation during the lifespan, implying that the animal lived close to areas with elevated amounts of the recognized trace metals; or (iii) derived from diagenetic processes. Some of the identified elements, such as Ca, Cu, Fe, and Zn, are indeed involved in physiological processes and, thus, may be truly endogenous. The diagenesis, led by the lithostatic pressure, could result in migration of fluids to lesser pressured beds, and ions may have become chelated with the organic matter ^21,31^. Moreover, some could also replace others with similar atomic radius, as is the case, for instance, of Rare Earth Elements, as well as Sr and Ca. For instance, some Rare Earth Elements can substitute Ca in apatite and calcite during diagenesis, where these elements mainly occupy the Ca I and Ca II sites ^25,33^.

**1.4. Raman Spectroscopy**

According to our results, the CPCA 3590 headcrest is mainly composed of calcium phosphate and eumelanin. These compounds were identified by diagnostic peaks among examined regions, which varied in their intensities. For instance, the PO_4_^3-^ bands occur at ca. 965 cm^-1^ ^29^, whereas the eumelanin between 1300 cm^-1^ and 1600 cm^-1^ ^34^. Furthermore, the phosphate spectra also exhibit bands of HCO_3_^–^ (from calcium carbonate), which usually occur between 1085 cm^-1^ and 1092 cm^-1^ ^35^. Thus, the overall spectra of the headcrest tissue consist of 282, 965, 1086 cm^-1^ bands (Fig. 2; Fig. S4), which are here assigned to the *v*_1_ and *v*_3_ vibrations of PO_4_^3+^ and CO_3_, respectively. Both compounds exhibit different peak intensities, and, for carbonate, bands are strong and narrow, whereas for phosphate they are weak and narrow. This feature is indicative of an ordered crystalline lattice, and the difference in intensity may suggest a stronger influence of the overlying matrix, which may mask PO_4_^3+^ scattering. The comparative analysis indicates that CPCA 3590 sample spectra are more related to hydroxyapatite due to the presence of 318 cm^-1^ and 1077 cm^-1^ bands.

The typical Raman spectrum of melanin is generally characterized by double and broad bands centred between ca. 1380 and 1585 cm^-1 36–38^. According to peak position, it can be assigned to different molecular bonds, such as out of plane deformation of O―H, C―OH from phenols, C―N stretching from pyrroles or indoles, among others (for details, see 17 and 22 and references therein). The broadness and relative intensity of bands may be caused by the irregular (less crystalline) arrangement of their carbon bonds ^40^. In CPCA 3590, Raman peaks are assigned to plane vibrations of C―C bonds, as well as stretching of C―OH (from COOH), C―N from pyrrole rings, and C―O from the phenolic group, as well as from indole ring vibration ^34,36–40^.

At the soft tissue, Raman spectra also exhibit two broad bands between ca. 1200 to 1650 cm^-1^ (Fig. 2, C; Fig. S4, B), and their identification varies largely among examined regions (Fig. S4, C and D). We kept the laser power at a minimum to avoid sample damaging, as a higher power or prolonged exposure time can produce, by blazing, the D and G bands of carbonaceous compounds, which are common for organic matter decomposition. This procedure also ensured that peaks between 1300 to 1600 cm^-1^ are not artefactual and are indeed derived from the original compound.

The fitting procedure performed using Gaussian function (*R^2^*=0.9539) revealed the exact position of the two bands (Fig. S4, B), which are centred at ca. 1336 cm^-1^ and 1567 cm^-1^ (Tab. S2). Although the signal is generally low as melanin is in trace amounts, these results strongly suggest that the spectra of CPCA 3590 are indeed derived from the vibration of eumelanin units.

In conclusion, the presence of peaks of carbonate indicates that the matrix has a significant contribution to the overall RS spectra; and this may be more pronounced in the melanin bands, which are broad and less intense. As a result, this influence could suggest that: (a) the laser beam has a deeper penetration than expected, and during the excitation, it gathers information from both fossil and the underlying matrix; and (b) melanin is present in low concentrations (trace amounts), possibly distributed in thin layers that are exceeded by the beam, which reaches the underlying matrix. Nevertheless, the identification of carbonates and phosphates strongly support the interpretation that microbodies were preserved by phosphatization as suggested previously ^19^. Therefore, the two broad bands observed unequivocally indicate that dark bands of *T. imperator* are composed of eumelanin.

**2. Detailed Methods**

**2.1. Synchrotron Radiation µX-Ray Fluorescence**

Examinations were performed at the UVX synchrotron light source of the Brazilian Synchrotron Light Laboratory from the Brazilian Centre for Research in Energy and Materials (LNLS-CNPEM), at the SR-μXRF beamline under the XRF-20170713 proposal. The experiment was performed using the white-beam mode with Iron (III) foil filters, the collimator of 3 mm, and the microbeam was provided by the KB system that produced a beam size of approximately 12 X 25 µm. Samples were placed onto aluminium sample-holders tilted 45° from the detector distant 20 cm. Examinations of small regions (ca. 1.0 to 5.2 mm) were carried out using the flyscan mode under 40 or 50 µm of step-size with an accumulation time of 0.1 seconds/point, with varying deadtime. The analysis generated various EDF and HDF archives, which were latter normalized and fitted, and its elements identified using the PyMCA software. Spectral and elemental maps were produced and saved as JPG and PNG, and further graphical processing was performed using Inkscape version 0.92.3 (2405546, 2018-03-11).

**2.2. Identification of PTCA and PTeCA**

To confirm the identification of PTCA and PTeCA in the alkaline peroxide oxidation mixture from CPCA 3590, the mixture was extracted with diethyl ether, dried as described previously ^41^. A 25 μL injection of CPCA 3590 at a concentration of 60-80 μM in a 75:25 mixture of LC grade methanol and water solution was injected onto an Agilent 1200 Series high-performance liquid chromatography system (HPLC, Agilent Technologies Inc.) and separated using a Ascentis Express 5 cm x 2.1 mm x 2.7 μm C_18_ column (Supelco Analytical) with a column temperature of 35°C. The HPLC was connected with a standard ESI interface to an Agilent Technologies 6224 MS-TOF to obtain high-resolution exact mass measurements. The LC-MS-TOF was operated at a flow rate of 0.17 mL/min using a linear gradient of 0.3% formic acid, 98% water, and 2% methanol (A) and 0.3% formic acid, 98% acetonitrile, and 2% water (B) as the mobile phase. The gradient program is provided in the table below (Table S3). The MS used an electrospray ionization (ESI) source in the negative mode. The results are shown in Figure S5.

**2.3. Raman Spectroscopy**

Prior to the Raman analysis, in order to eliminate modern contaminants during transport, the small fragment was washed in ethanol and left to dry inside a partially closed petri dish at ambient temperature and humidity. The sample was handled using disposable powder-free gloves. This washing procedure was repeated three times before the fragment was placed onto sterile glass slides in the confocal micro-Raman In Via Renishaw equipped with, He-Ne monochromatic lasers with 633 and 785 nm, detector CCD and spectral resolution of 4 cm^-1^. Spectra were collected with a spectral range between 200-2000 cm^-1^, with 0.05 to 1% laser power and an exposure time from 1 to 10 seconds, with an average of 20 accumulations. The analysis was made both in mapping and point-and-shoot modes, and examinations were carried out with sample devoid of any type of coating. In order to distinguish the materials, the spectrum was obtained using WiRE 4.1 and using Origin 8 (OriginLab). Spectra were normalized, and smoothing processing was performed using the Savitzky-Golay filter. Subtract baseline was performed using Fityk 0.9.8 ^42^, and deconvolutions were carried out using the Gaussian function with a spectral range between 1200 to 1700 cm^-1^, where only correlation values below *R^2^*=0.98 were considered. For spectra comparison, we examined the synthetic melanin from the oxidation of tyrosinase by hydrogen peroxide (M8631) and natural melanins from *Sepia officinalis* (M2649) purchased from Sigma-Aldrich Co. (Saint Louis - MO, USA). We also used the standard mineral spectra from the database of the RRUFF Project ^43^. The RS equipment (FAPESP 2012/18936-0) is housed in the Research Unit of Astrobiology of the University of São Paulo (NAP/Astrobio, PRP-USP) currently at the TGM beamline (Toroidal Grating Monochromator) of the Brazilian Synchrotron Light Laboratory (LNLS) of the National Centre for Energy and Materials Research.

**Cited References**

1. Coimbra, J. C., Arai, M. & Carreño, A. L. Biostratigraphy of Lower Cretaceous microfossils from the Araripe basin, northeastern Brazil. *Geobios* **35**, 687–698 (2002).

2. Assine, M. L. *et al.* Sequências deposicionais do Andar Alagoas da Bacia do Araripe, Nordeste do Brasil. *Bol. Geociencias da Petrobras* **22**, 3–28 (2014).

3. Neumann, V. H. M. L. & Cabrera, L. Características Hidrogeológicas gerais, mudanças de salinidade e caráter endorréico do sistema lacustre Cretáceo do Araripe, NE Brasil. *Rev. Geol.* **15**, 43–54 (2002).

4. Neumann, V. H., Borrego, A. G., Cabrera, L. & Dino, R. Organic matter composition and distribution through the Aptian-Albian lacustrine sequences of the Araripe Basin, northeastern Brazil. *Int. J. Coal Geol.* **54**, 21–40 (2003).

5. Heimhofer, U. *et al.* Deciphering the depositional environment of the laminated Crato fossil beds (Early Cretaceous, Araripe Basin, North-eastern Brazil). *Sedimentology* **57**, 677–694 (2010).

6. Neumann, V. H. & Cabrera, L. Significance and genetic interpretation of the sequential organization of the Aptian-Albian. *An. Acad. Bras. Cienc.* **72**, 607–608 (2000).

7. Heimhofer, U. & Martill, D. M. The sedimentology and depositional environment of the Crato Formation. in *The Crato Fossil Beds of Brazil: Window into an Ancient World* (eds. Martill, D. M., Bechly, G. & Loveridge, R. F.) 44–62 (Cambridge University Press, 2007). doi:10.1017/CBO9780511535512.005

8. Silva, V. R. *et al.* First record of Neithea from the Aptian Crato Formation, Araripe Basin, Brazil, and it’s significance. in *Boletim de resumos do XXV Congresso Brasileiro de Paleontologia* 320–320 (Sociedade Brasileira de Paleontologia, 2017).

9. Catto, B., Jahnert, R. J., Warren, L. V., Varejão, F. G. & Assine, M. L. The microbial nature of laminated limestones: Lessons from the Upper Aptian, Araripe Basin, Brazil. *Sediment. Geol.* **341**, 304–315 (2016).

10. Warren, L. V. *et al.* Stromatolites from the Aptian Crato Formation, a hypersaline lake system in the Araripe Basin, northeastern Brazil. *Facies* **63**, 1–19 (2017).

11. Osés, G. L. *et al.* Deciphering pyritization-kerogenization gradient for fish soft-tissue preservation. *Sci. Rep.* **7**, (2017).

12. Osés, G. L. *et al.* Deciphering the preservation of fossil insects: a case study from the Crato Member, Early Cretaceous of Brazil. *PeerJ* **4**, e2756 (2016).

13. Varejão, F. G. *et al.* Exceptional preservation of soft tissues by microbial entombment: Insights into the taphonomy of the Crato Konservat-Lagerstätte. *Palaios* **34**, 331–348 (2019).

14. Pinheiro, F. L., Horn, B. L. D., Schultz, C. L., Andrade, J. A. F. G. & Sucerquia, P. A. Fossilized bacteria in a Cretaceous pterosaur headcrest. *Lethaia* **45**, 495–499 (2012).

15. Prado, G. M. E. M., Anelli, L. E., Petri, S. & Romero, G. R. New occurrences of fossilized feathers: systematics and taphonomy of the Santana Formation of the Araripe Basin (Cretaceous), NE, Brazil. *PeerJ* **4**, e1916 (2016).

16. Li, Q. *et al.* Reconstruction of Microraptor and the Evolution of Iridescent Plumage. *Science (80-. ).* **335**, 1215–1219 (2012).

17. Schweitzer, M. H., Lindgren, J. & Moyer, A. E. Melanosomes and ancient coloration re-examined: A response to Vinther 2015 (DOI 10.1002/bies.201500018). *BioEssays* **37**, 1174–1183 (2015).

18. Solano, F. Melanins: Skin Pigments and Much More—Types, Structural Models, Biological Functions, and Formation Routes. *New J. Sci.* **2014**, 1–28 (2014).

19. Pinheiro, F. L., Fortier, D. C., Schultz, C. L., Andrade, J. A. F. G. & Bantim, R. A. M. New information on the pterosaur Tupandactylus imperator, with comments on the relationships of Tapejaridae. *Acta Palaeontol. Pol.* **56**, 567–580 (2011).

20. Wogelius, R. A. *et al.* Trace Metals as Biomarkers for Eumelanin Pigment in the Fossil Record. *Science (80-. ).* **333**, 1622–1626 (2011).

21. Bergmann, U. *et al.* Archaeopteryx feathers and bone chemistry fully revealed via synchrotron imaging. *Proc. Natl. Acad. Sci.* **107**, 9060–9065 (2010).

22. Bergmann, U., Manning, P. L. & Wogelius, R. A. Chemical mapping of paleontological and archeological artifacts with synchrotron X-rays. *Annu. Rev. Anal. Chem. (Palo Alto. Calif).* **5**, 361–89 (2012).

23. Edwards, N. P. *et al.* Mapping prehistoric ghosts in the synchrotron. *Appl. Phys. A Mater. Sci. Process.* **111**, 147–155 (2013).

24. Gueriau, P. *et al.* Trace Elemental Imaging of Rare Earth Elements Discriminates Tissues at Microscale in Flat Fossils. *PLoS One* **9**, e86946 (2014).

25. Gueriau, P. & Bertrand, L. Deciphering Exceptional Preservation of Fossils Through Trace Elemental Imaging. *Micros. Today* **23**, 20–25 (2015).

26. Gueriau, P., Jauvion, C. & Mocuta, C. Show me your yttrium, and I will tell you who you are: implications for fossil imaging. *Palaeontology* **61**, 981–990 (2018).

27. Gueriau, P., Bernard, S. & Bertrand, L. Advanced Synchrotron Characterization of Paleontological Specimens. *Elements* **12**, 45–50 (2016).

28. Edwards, N. P. *et al.* Elemental characterisation of melanin in feathers via synchrotron X-ray imaging and absorption spectroscopy. *Sci. Rep.* **6**, 1–10 (2016).

29. Morris, M. D. & Mandair, G. S. Raman assessment of bone quality. *Clin. Orthop. Relat. Res.* **469**, 2160–2169 (2011).

30. Hong, L., Liu, Y. & Simon, J. D. Binding of Metal Ions to Melanin and Their Effects on the Aerobic Reactivity. *Photochem. Photobiol.* **80**, 477 (2004).

31. Egerton, V. M. *et al.* The mapping and differentiation of biological and environmental elemental signatures in the fossil remains of a 50 million year old bird. *J. Anal. At. Spectrom.* **30**, 627–634 (2015).

32. Manning, P. L. *et al.* Synchrotron-based chemical imaging reveals plumage patterns in a 150 million year old early bird. *J. Anal. At. Spectrom.* **28**, 1024–1030 (2013).

33. Chen, H. & Stimets, R. W. Fluorescence of trivalent neodymium in various materials excited by a 785 nm laser. *Am. Mineral.* **99**, 332–342 (2014).

34. Perna, G., Lasalvia, M. & Capozzi, V. Vibrational spectroscopy of synthetic and natural eumelanin. *Polym. Int.* **65**, 1323–1330 (2016).

35. Gunasekaran, S., Anbalagan, G. & Pandi, S. Raman and infrared spectra of carbonates of calcite structure. *J. Raman Spectrosc.* **37**, 892–899 (2006).

36. Huang, Z. *et al.* Raman spectroscopy of in vivo cutaneous melanin. *J. Biomed. Opt.* **9**, 1198 (2004).

37. Galván, I. *et al.* Raman spectroscopy as a non-invasive technique for the quantification of melanins in feathers and hairs. *Pigment Cell Melanoma Res.* **26**, 917–923 (2013).

38. Galván, I., Jorge, A., Solano, F. & Wakamatsu, K. Vibrational characterization of pheomelanin and trichochrome F by Raman spectroscopy. *Spectrochim. Acta Part A Mol. Biomol. Spectrosc.* **110**, 55–59 (2013).

39. Perna, G. Vibrational Characterization of Synthetic Eumelanin by Means of Raman and Surface Enhanced Raman Scattering. *Open Surf. Sci. J.* **5**, 1–8 (2013).

40. Galván, I. & Jorge, A. Dispersive Raman spectroscopy allows the identification and quantification of melanin types. *Ecol. Evol.* **5**, 1425–1431 (2015).

41. Glass, K. E. *et al.* Direct chemical evidence for eumelanin pigment from the Jurassic period. *Proc. Natl. Acad. Sci.* **109**, 10218–10223 (2012).

42. Wojdyr, M. Fityk : a general-purpose peak fitting program. *J. Appl. Crystallogr.* **43**, 1126–1128 (2010).

43. Lafuente, B., Downs, R. T., Yang, H. & Stone, N. *The power of databases: The RRUFF project*. *Highlights in Mineralogical Crystallography* (2016). doi:10.1515/9783110417104-003

44. Clarke, J. A. *et al.* Fossil evidence for evolution of the shape and color of penguin feathers. *Science (80-. ).* **330**, 954–957 (2010).


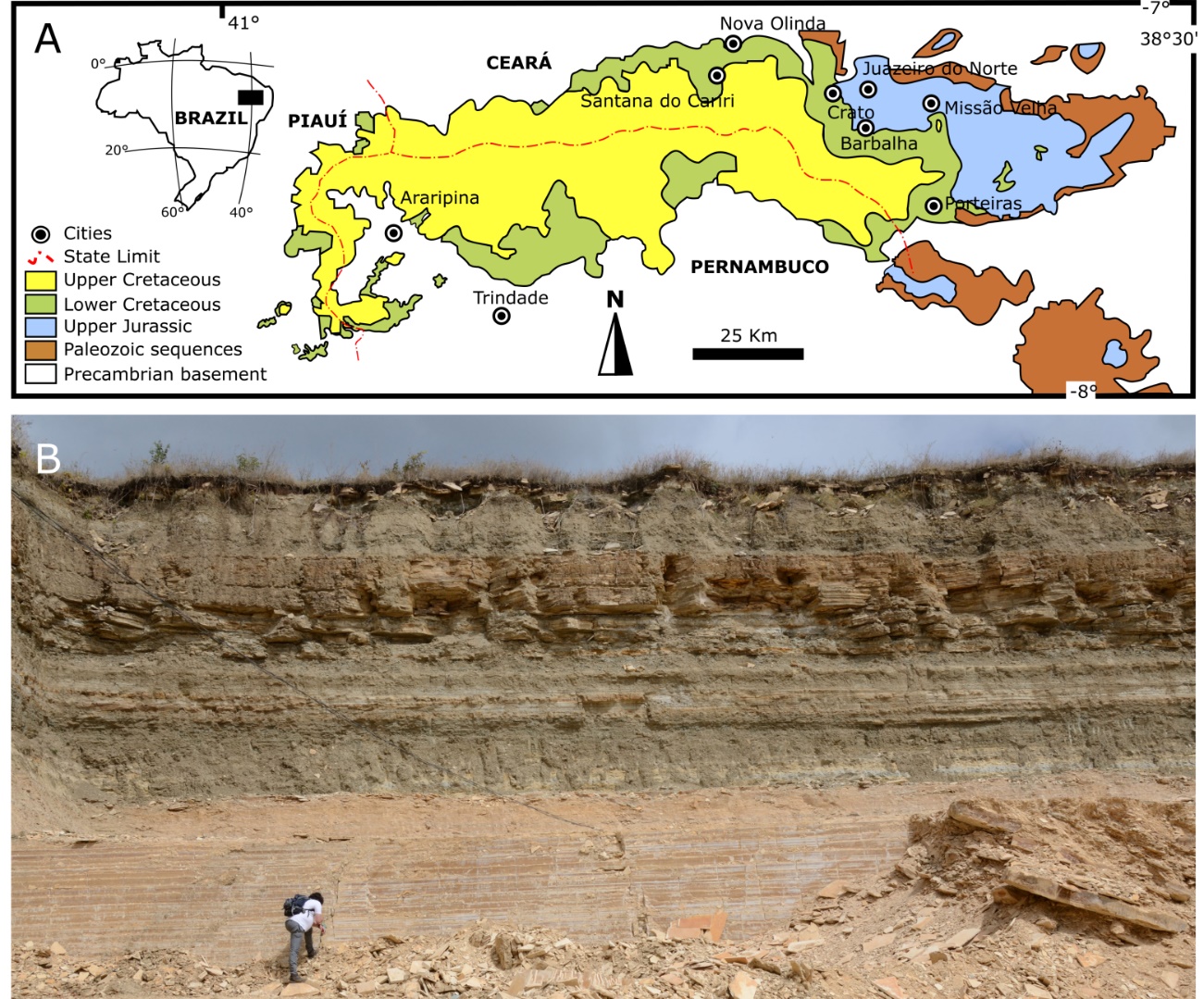


Figure S1. Araripe Basin and Crato Formation (A) Location of the Araripe Basin, showing the sedimentary sequences distribution, in which the Crato Formation occur at N-SE part of the green limit. (B) Crato Formation outcrop, exhibiting thick sequences of laminated limestones interbedded by greenish shales and sandstones of the Nova Olinda member at the *Triunfo* Quarry located between Crato and Santana do Cariri cities.


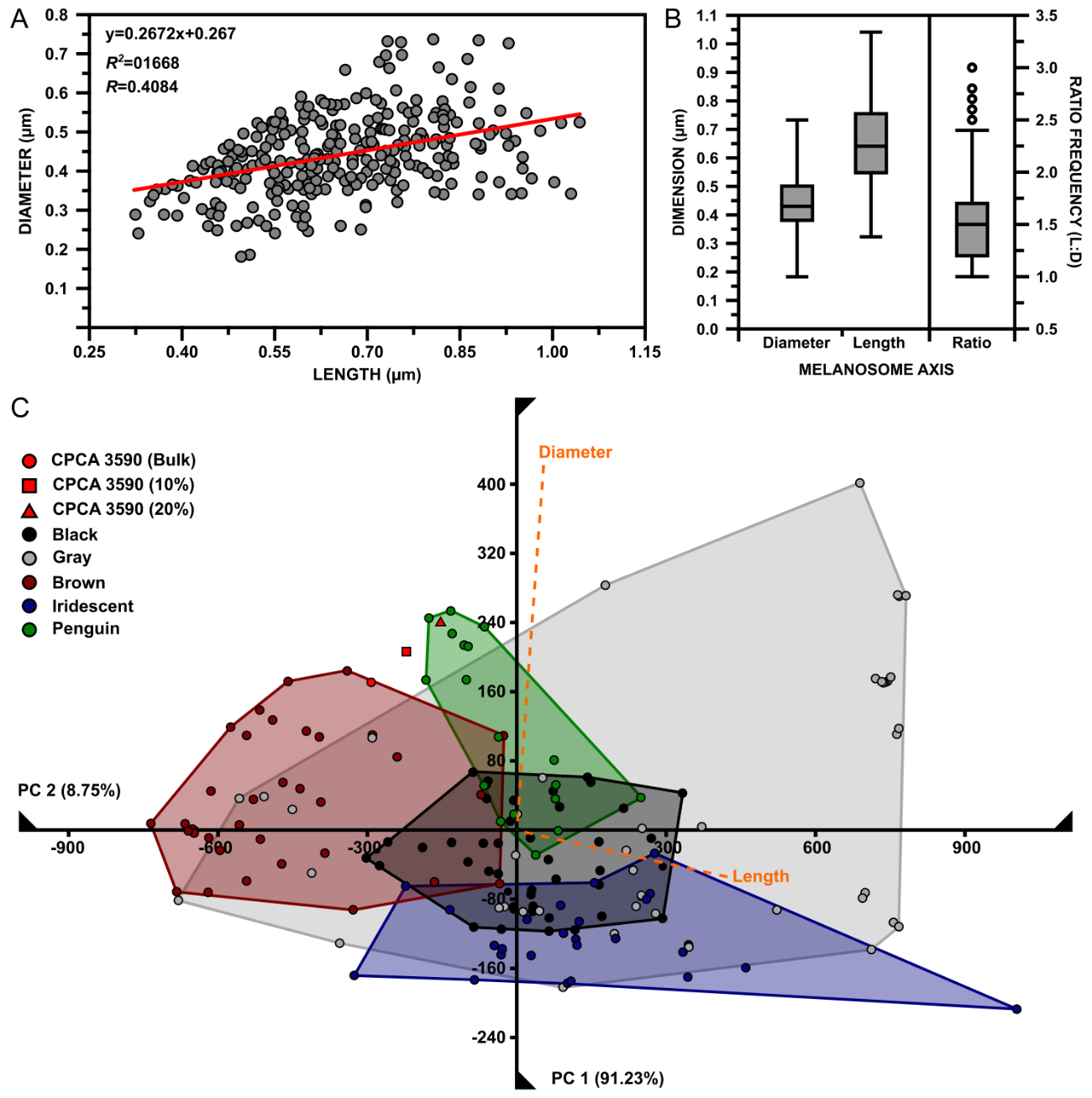


**Figure S2.** Statistical analysis of CPCA 3590 melanosomes (n=331). (**A**) Scatter plot and (**B**) boxplot of the melanosome axes (length and diameter) exhibiting a weak correlation (r=0.4084; *R^2^*=0.1668) and the overall dimension of the melanosomes. Mean sizes that fall at around 441 nm in diameter, 652 µm in length and 1.5 in ratio. (**C**) Principal component analysis of the Li et al. ^16^ database (melanosome shape/colour) showing that diameter and length place the *T. imperator* microbodies close to melanosomes of brown and penguin colours (i.e. blue and black hues, see Clarke et al. ^44^.


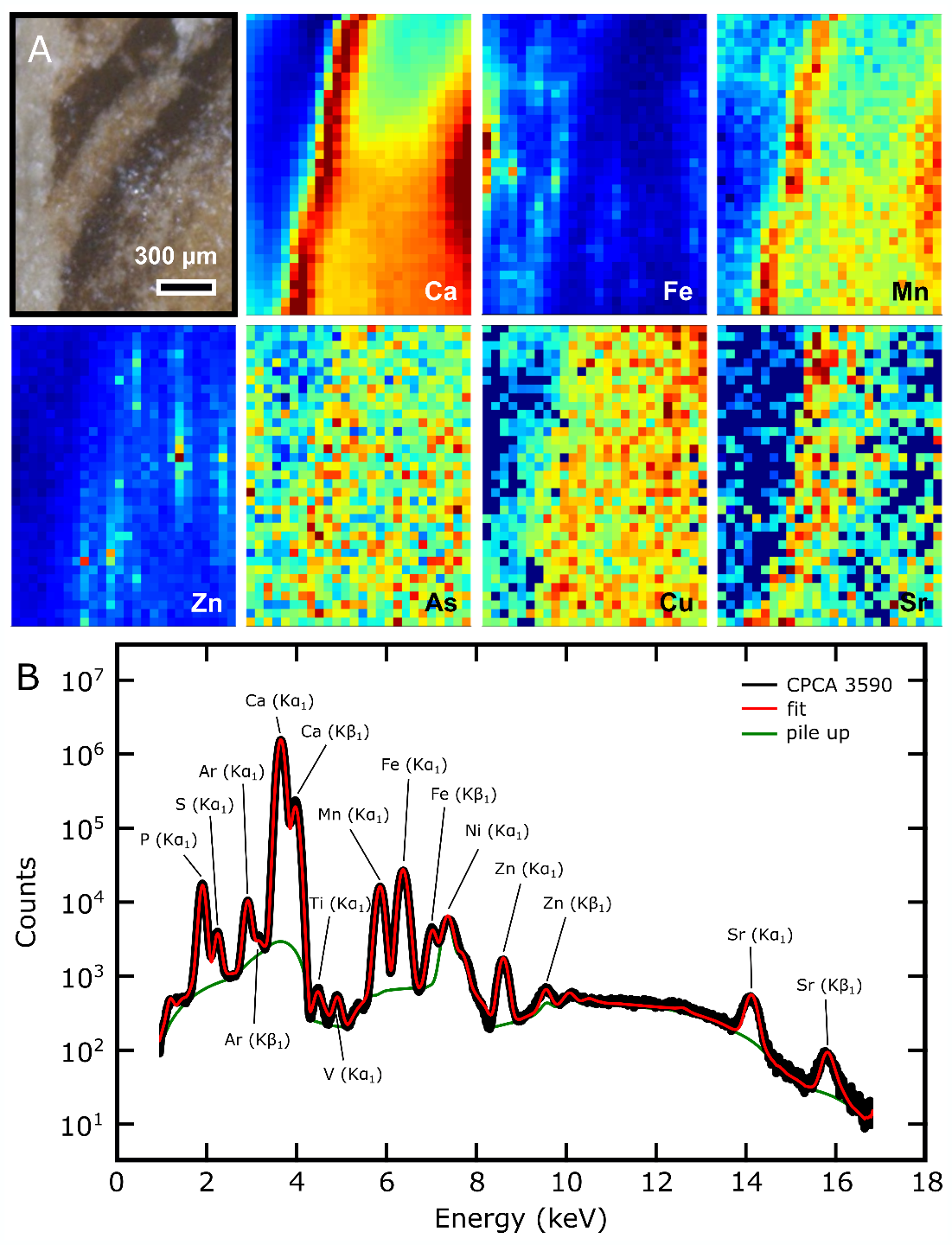


**Figure S3.** SR-µXRF elemental analysis of CPCA 3590 tissue. (**A**) SR-μXRF map of the headcrest tissue showing elements with good spatial correlation. Colors indicate the relative intensity, where the higher are shown in red while the lower in blue. (**B**) The fitted spectra of the region in (A) exhibiting the presence of other elements that occur without significant intensity and spatial correlation.


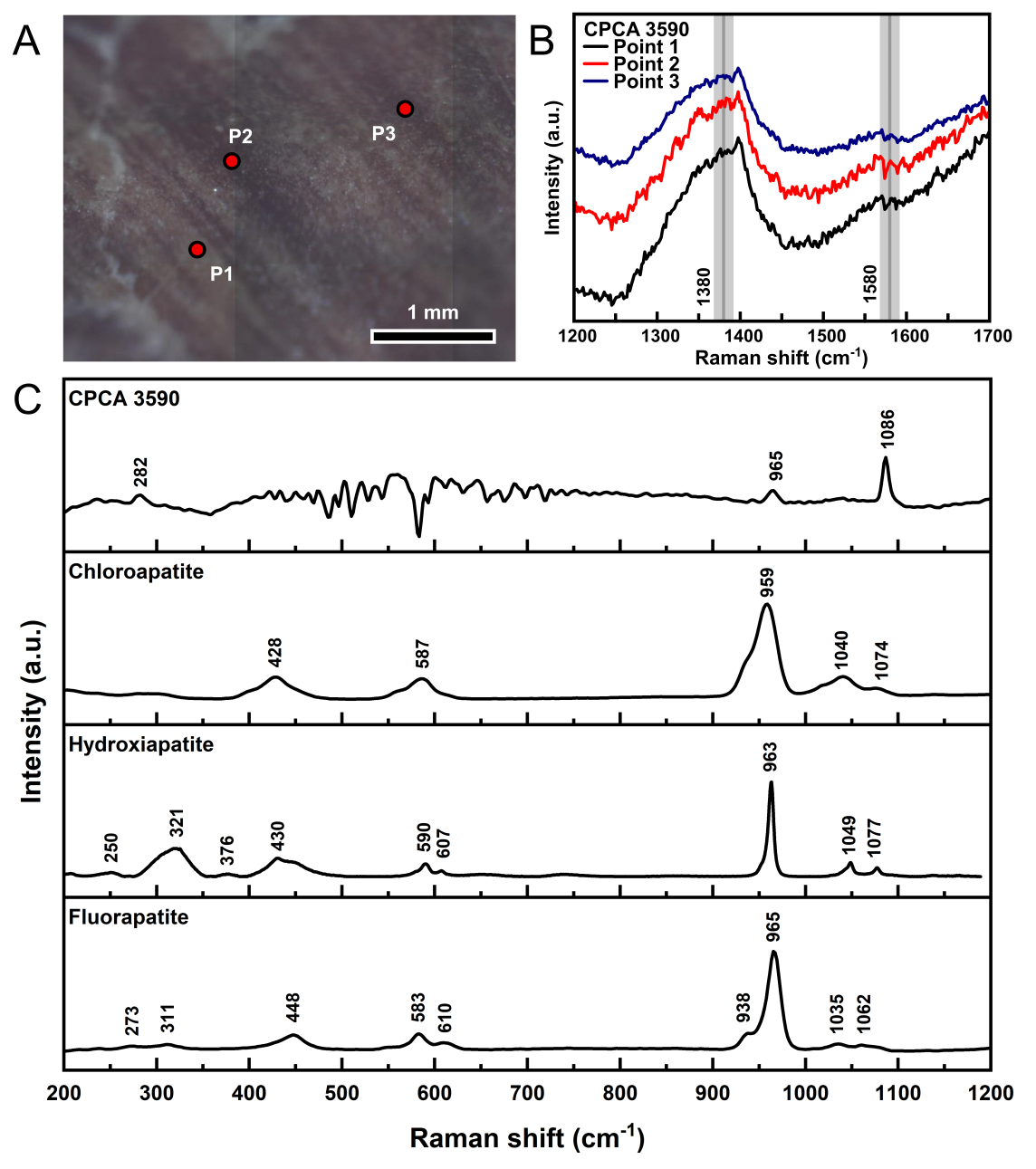


**Figure S4.** Raman spectra of *Tupandactylus imperator* headcrest. (A) Spectra from the banded tissue showing the three points analysed, where the P1 was carried out at the lighter portion, whereas P2 and P3 measures were taken from darker spots. (B) Spectra from the three points seen in (A) exhibiting the two bands of different intensities indicating melanin’s presence; the first band centred at about 1380 cm^-1^ is pronounced, whereas the second band (ca. 1580 cm^-1^) is almost undetectable. Grey lines and shadows represent the expected bands of eumelanin and ±10 cm^-1^ range, respectively. (C) FTRaman spectra from the bony part of the headcrest showing similar bands with standard calcium phosphates from the RRUFF project.


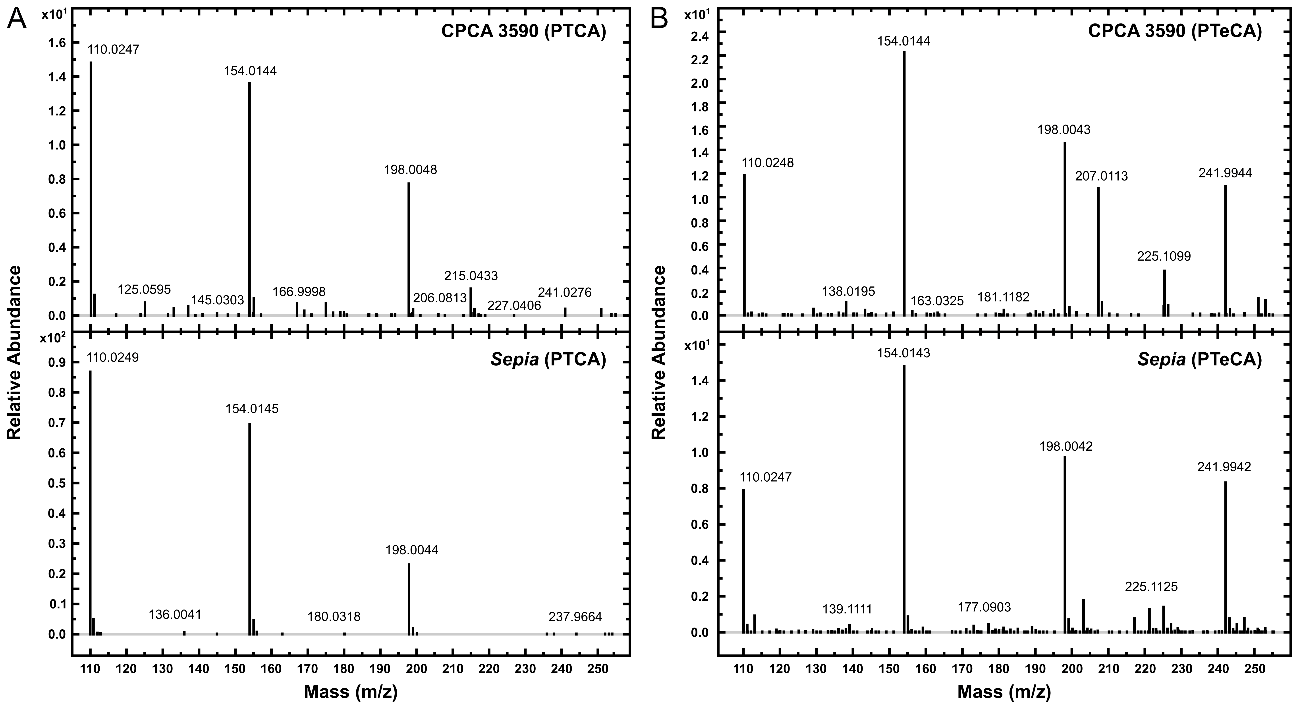


**Figure S5.** Mass spectra of the degradation products of the CPCA 3590 and Sepia melanin, where’s (**A**) is from PTCA and (**B**) from PTeCA. Structures associated with the peaks of the fragments formed from the parent ion, the ion with the greatest mass in each of the spectrum above, are indicated.

Table S1. Average numbers of the measures (n=331) of entire melanosomes microbodies of CPCA 3590 specimen, with 10% and 20% corrected values. The diameter, length and standard deviation (SD) variables are all in nm.

| ***CPCA 3590*** | ***Length ± SD*** | ***Length***  ***CV*** | ***Length Skew*** | ***Diameter***  ***± SD*** | ***Diameter***  ***CV*** | ***Diameter***  ***Skew*** | ***Ratio*** | ***Ratio CV*** | ***Ratio***  ***Skew*** | ***Density*** |
| --- | --- | --- | --- | --- | --- | --- | --- | --- | --- | --- |
| Bulk | 652.937  ± 147.513 | 0.2 | 0.2 | 441.420 ± 96.499 | 0.2 | 0.4 | 1.52 | 0.3 | 1.0 | 4.0 |
| 10% | 718.284 ± 162.278 | 0.2 | 0.2 | 485.589 ± 106.161 |  |  |  |  |  |  |
| 20% | 783.553 ± 177.008 | 0.2 | 0.2 | 529.737 ± 115.811 |  |  |  |  |  |  |

**Table S2.** Results of fitting using a Gaussian function for the headcrest tissue. DF - degrees of freedom; RCS - reduced chi-squared; A-R^2^ – Adjusted R-Square.

| ***Band*** | ***Area*** | ***Center*** | ***FWHM*** | ***DF*** | ***R^2^*** | ***RCS*** | ***A-R^2^*** |
| --- | --- | --- | --- | --- | --- | --- | --- |
| Band 1 | 69167.71 | 940.85 | 83.82 | 658 | 0.9852 | 69206.60921 | 0.9847 |
| Band 2 | 223739.21 | 1140.59 | 193.61 |  |  |  |  |
| Band 3 | 398184.71 | 1327.88 | 80.02 |  |  |  |  |
| Band 4 | 526396.02 | 1407.33 | 227.12 |  |  |  |  |
| Band 5 | 866003.25 | 1573.35 | 112.53 |  |  |  |  |
| Band 6 | 8592.32 | 1693.57 | 15.58 |  |  |  |  |
| Band 7 | 278276.99 | 1780.72 | 352.63 |  |  |  |  |
| Band 8 | 17204.12 | 1892.48 | 54.00 |  |  |  |  |

**Table S3.** Gradient program for LC-MS-TOF analysis of CPCA 3590.

| ***Time (min)*** | ***%A*** | ***%B*** |
| --- | --- | --- |
| 0 | 100 | 0 |
| 4 | 100 | 0 |
| 12 | 85 | 15 |
| 18 | 40 | 60 |
| 22 | 40 | 60 |
| 28 | 100 | 0 |
| 29 | 100 | 0 |
